# Intracranial Solitary Fibrous Tumor: A Case of a 21-Year-Old Male With Olfactory Hallucination

**DOI:** 10.7759/cureus.60104

**Published:** 2024-05-11

**Authors:** Jackson E Rudolph, Guerard P Grice, Michael H Lawless

**Affiliations:** 1 Medicine, Uniformed Services University of the Health Sciences, Bethesda, USA; 2 Neuropathology, Naval Medical Center San Diego, San Diego, USA; 3 Neurosurgery, Naval Medical Center San Diego, San Diego, USA

**Keywords:** malignant solitary fibrous tumor, olfactory disturbance, intracranial solitary fibrous tumor, meningioma-like lesion, solitary fibrous tumor (sft)

## Abstract

Meningeal solitary fibrous tumors (SFTs) are a rare central nervous system neoplastic process, resulting in frequent misdiagnosis as meningioma prior to pathologic analysis. Appropriate diagnosis is essential to lowering morbidity and mortality, as Grade II or III SFTs are aggressive neoplasms that possess metastatic potential. The existing data may suggest that intracranial SFTs primarily afflict those in their fourth through sixth decades of life. However, we present the case of a patient outside this demographic presenting with symptoms that we were unable to identify in any prior reports. A 21-year-old male in the United States Navy presented to the emergency department (ED) with a two-month history of progressive headaches, leading to nausea and emesis. The patient also endorsed a daily incidence of the same olfactory hallucination followed by several minutes of palpitations, flushing, and dizziness. His neurologic exam was unremarkable, but imaging in the ED revealed a large mass abutting the right medial sphenoid wing. The radiographic appearance of the mass with a dural tail led to a preoperative diagnosis of meningioma. However, pathologic analysis following gross total resection identified the mass as an SFT. A brief literature review complementary to this case underscored the high variability of intracranial SFT case presentations with a relative scarcity of epidemiologic data due to rarity. This review identified that it was common to initially diagnose SFTs as meningioma, similar to this particular case. This emphasizes the importance of an appropriate pathologic diagnosis. This case adds to the existing literature as anecdotal evidence of SFT occurring in a young patient and a unique symptom profile most notable for olfactory hallucination and dysautonomia as features of focal seizure.

## Introduction

Solitary fibrous tumors (SFTs) are spindle cell neoplasms first identified and most commonly found in the pleura [1]. Since that first description in 1931, SFTs have been diagnosed in numerous soft tissues as well as the mediastinum, pericardium, upper respiratory tract, peritoneum, and more. Central nervous system (CNS) manifestations of SFTs are especially rare and are most often found along the meninges, spinal dura, at the cerebellopontine angle, and in the parasagittal and intraventricular regions [1]. Following WHO reclassification, SFTs were combined in a group with hemangiopericytomas as both are characterized by the *NAB2-STAT6* gene fusion, although they differ in biologic behavior [2]. SFT grading ranges from I to III based on cellularity and mitotic activity, with Grades II and III being associated with a higher risk of malignancy [2].

The reliable epidemiology of these tumors is difficult to assess due to their rarity. One study calculated an age-adjusted incidence of CNS SFT at 3.77 per 10,000,000 [3]. The same study identified a median age at diagnosis of 54 and a slight female predominance in approximately 53% of all cases, though not statistically significant. Additionally, the study found that supratentorial location was more common than infratentorial position [3]. Regarding tumor location, the middle cranial fossa seems to be exceptionally rare, given that a literature review spanning from 1997 to 2021 was only able to isolate 16 publications featuring 19 case studies of SFTs in this anatomic region [4]. Our case is that of an SFT abutting the right medial sphenoid wing, which constitutes part of the anterior border of the middle cranial fossa. This anatomic location and an unusual constellation of symptoms make our case unique.

## Case presentation

A 21-year-old male active duty service member presented to the emergency department with a two-month history of progressive headache leading to nausea and emesis that prompted him to seek care. He reported a several-month history of episodes in which he experienced the same “odd smell” regardless of his surroundings with no apparent stimuli. This olfactory hallucination was immediately followed by chest pressure, palpitations, flushing, and dizziness lasting 5-10 minutes per episode. The longest such episode lasted one hour, and none of the episodes featured loss of consciousness or convulsions. He also endorsed intermittent headaches on a similar timeline, but none as severe as the headache at the time of presentation.

His neurologic exam was unremarkable, followed by a radiographic work-up that revealed an intradural, extra-axial mass based on the right medial sphenoid wing measuring 4.5 cm in diameter with surrounding vasogenic edema in the basal frontal and medial temporal lobes. The mass abutted the superior orbital fissure but did not appear to invade the fissure or the optic canal. Given the finding of this large mass with vasogenic edema and presentation with concomitant olfactory hallucinations and autonomic symptoms consistent with focal seizures, surgical resection was indicated to relieve the mass effect, confirm the pathologic diagnosis, and control seizures.

**Figure 1:**
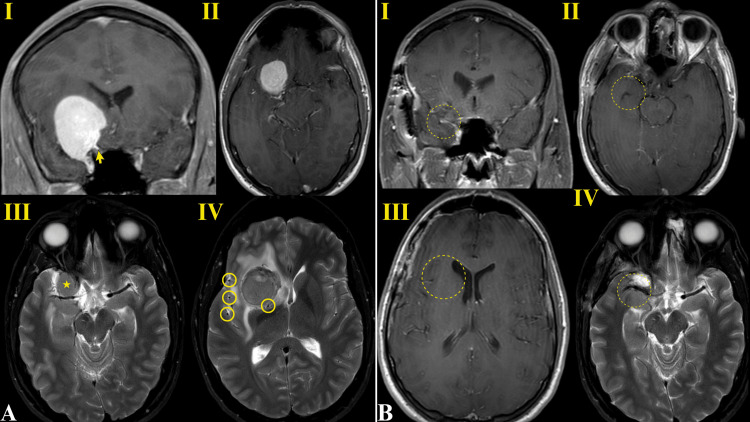
Preoperative (A) and postoperative (B) MRI findings The preoperative imaging is most notable for avid homogenous enhancement with a dural tail on T1 postgadolinium sequences identified by the yellow arrow in A_I_ and mild peripherally prominent flow voids on T2 sequences highlighted by the yellow circles in A_IV_, which are more consistent with typical meningioma appearance. A_II_ provides an axial view of the mass with the T1 sequence, and the star in A_III_ denotes the location of the mass at the level of the optic chiasm. The postoperative imaging was obtained three months following surgery and did not reveal any sign of residual or recurrent tumor. The approximated previous location of the tumor is displayed as a dashed circle in each panel in B_I-IV_. MRI: magnetic resonance imaging.

Based on radiographic findings of a dural tail along the right sphenoid wing and a generally homogenous appearance, our leading preoperative diagnosis was meningioma (Figure 1). A preoperative ophthalmologic examination revealed normal vision in the right eye and no visual field loss.

The surgical approach was a right-sided stereotactic pterional craniotomy, utilizing subfascial temporalis dissection for the preservation of the frontalis nerve. The orbital roof was drilled flush and the greater sphenoid wing to the lateral meningo-orbital band. After opening the dura and identifying the superior pole of the tumor, the tumor capsule was coagulated and incised, followed by internal debulking with an ultrasonic aspirator. The medial attachment of the tumor to the dura overlying the sphenoid wing was inspected and coagulated for further tumor devascularization. After incising and dissecting the posterior aspect of the tumor due to its size, the anteromedial aspect of the tumor was mobilized using a series of bipolar and microdissections. There was a mild invasion of the pia on the basal frontal lobe, which was dissected from the tumor using cottonoid patties. The optico-carotid cistern was identified and preserved as well. Following the completion of standard dissection, the tumor was removed from the resection cavity. A small vessel supplying the tumor was torn during removal, which was promptly identified and coagulated. The case was uncomplicated, achieving gross total resection with an estimated blood loss of 300 mL. Postoperatively, the patient did not experience any further olfactory disturbance or dysautonomia consistent with his prior seizure episodes.

**Figure 2:**
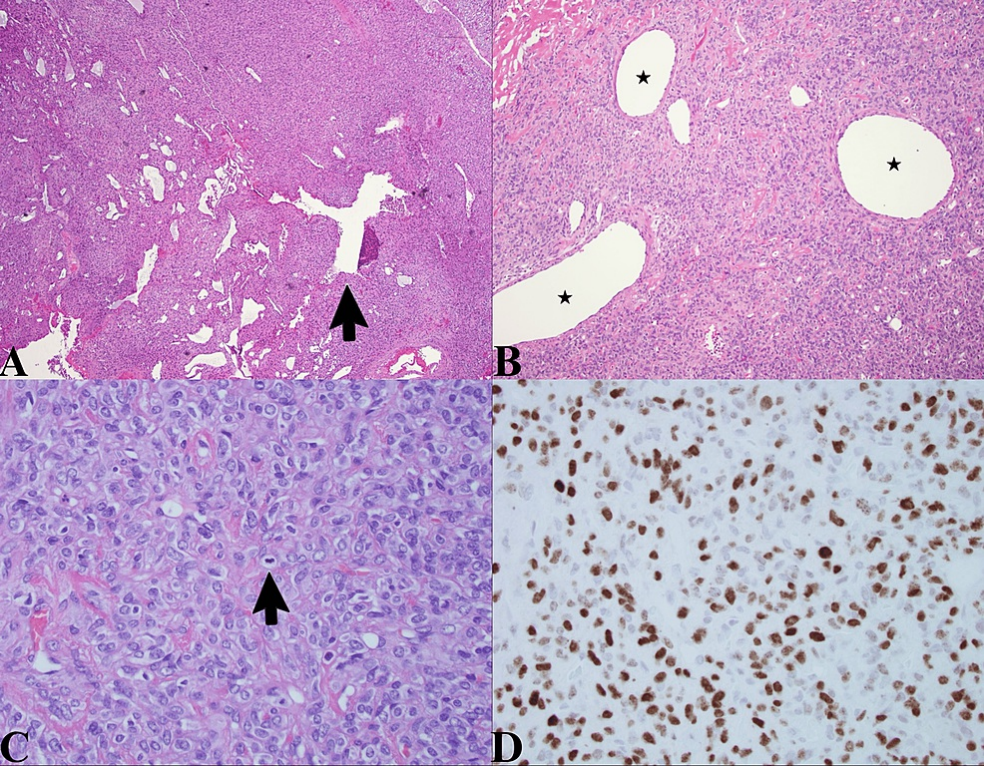
Histologic analysis A. This low-power view shows the hyperchromatic nuclei of the highly cellular tumor and the thin-walled, gaping blood vessels with an occasional “staghorn-type” vessel (arrow). B. At intermediate power, less cellular areas show more interstitial collagen as well as gaping, thin-walled blood vessels labeled with stars. C. At high power, the cytological features of the tumor cells are appreciated, and there are two mitoses in this field (one at the tip of the arrow and one below the butt of the arrow). D. This image depicts diffuse STAT6 brown staining of the tumor cell nuclei.

The specimen was sent to pathology, who reported that hematoxylin and eosin-stained sections showed a highly cellular tumor with many “gaping,” thin-walled blood vessels in which an occasional prototypical “staghorn-type” blood vessel was observed (i.e., a branching vessel with an antler-like configuration; Figure 2A). These vessels contrast with the thick-walled, hyalinized blood vessels that are more typical of meningiomas. Somewhat less cellular areas of the tumor also displayed the thin-walled gaping vessels, but there was a greater amount of pink interstitial collagen between the tumor cells (Figure 2B). At high power, many mitotic figures were noted, tumor cells exhibiting round-to-oval vesicular nuclei with visible nucleoli (Figure 2C). The mitotic count exceeded five mitoses per 10 high-power fields, but no necrosis was identified. A Ki-67 immunostain revealed a proliferation index of up to 15%. The tumor cells stained diffusely with CD99 and BCL-2. The glial fibrillary acidic protein (GFAP), epithelial membrane antigen (EMA), and CD34 immunostains were negative in the tumor cells. Most importantly, there is diffuse nuclear staining with STAT6, which, in the setting of the previously described histologic features, is pathognomonic for an SFT (Figure 2D). Positive STAT6 staining has been identified in as much as 98% of SFT cases [5]. The observed mitotic count and the absence of necrosis correspond to a Grade II tumor.

The grading of this tumor led to a postoperative plan including adjuvant proton radiation to mitigate the risk of recurrence, a full-body positron emission tomography (PET) scan to rule out extracranial metastases, and a three-month postoperative MRI that was not suspicious for residual or recurrent tumor. He returned to the clinic four weeks post-op, only complaining of mild residual headaches responsive to episodic self-dosing of over-the-counter Tylenol. The focal seizures featuring olfactory hallucination and vasomotor symptoms had completely resolved. He was placed on three months of limited duty to recover from surgery and resumed full military service after that period.

## Discussion

As mentioned above, our leading diagnosis before receiving the final pathology report was meningioma, a very common misdiagnosis for intracranial SFTs, as meningiomas are substantially more common and have a similar appearance on MRI. Among 19 cases of SFTs in the middle cranial fossa reviewed by Maiuri et al., meningioma was the only preoperative diagnosis reported (15 cases were “not specified," and four admitted to suspecting meningioma) [4]. Another recently published review of 21 craniospinal SFT cases performed by Piscopo et al. reported that 10 of their cases received an initial diagnosis of meningioma on radiological examination [6]. An interesting radiologic difference described in that case series is that the lesions were generally described as heterogenous, whereas the lesion in our patient was more homogenous in appearance. Diagnosing SFT appropriately is paramount because, although these tumors are localized at presentation, they carry a significant risk of recurrence and metastasis [6]. Therefore, maximal safe gross total resection is the goal of treatment with the use of adjuvant radiation for high-grade lesions to minimize this risk [6].

In addition to the importance of SFTs commonly being misdiagnosed as meningiomas on imaging, the key features of this case are the age at diagnosis and the notable symptoms of olfactory hallucination and autonomic dysfunction. None of the aforementioned 19 cases of SFT in the middle cranial fossa were identified in a patient as young or having the same constellation of symptoms as what we describe here. All but five of these cases were featured in a literature review by Gopakumar et al. that was published about 16 months prior and described 14 out of 58 (24%) intracranial SFT cases located on the middle cranial fossa or the sphenoid bone [7]. This larger review identified five patients who were diagnosed at a younger age than our patient, ranging from 10 days to 20 years, but none of their tumors were located in the middle cranial fossa [7]. More broadly, our literature review only found one case featuring a patient younger than ours with SFT of the middle cranial fossa/sphenoid wing, a 20-year-old female with a right-sided mass who presented with headache, nausea, vomiting, visual field defects, and seizures [8]. These examples, including our case, are important outliers considering a growing consensus that SFTs most commonly afflict the middle-aged. The population-based study by Kinslow et al., which serves as one of the few sources of epidemiologic data regarding CNS SFTs, calculated a median age of 54 [3]. Four different case series and single-institution retrospective analyses reported mean ages of 55.4, 46.4, 43, and again 43, with an additional case series reporting a median age of 45.5 [6,9-12].

Aside from the rarity of this diagnosis at this patient’s age, as it seems to be one of only a few dozen cases in the literature, this patient’s unique presentation should be appreciated. Headache is a common symptom of intracranial SFTs. In a study conducted by Clarencon et al., which included nine cases of these tumors, eight patients reported headache as a presenting symptom, similar to our patient [9]. An even larger study of 38 patients with intracranial SFTs corroborated this finding, as 27 of them endorsed headache [12]. However, this outcome is to be expected for an intracranial mass and lacks particular characteristics. In our review of the literature, we appreciated a wide variety of mass-effect sequelae specific to the cortical region or cranial nerves near the mass. While our patient’s focal seizures were likely attributable to mass effect and peritumoral edema, we did not identify any cases that described the particular combination of autonomic symptoms and olfactory hallucination that we observed [13]. The case series conducted by Liu et al. identified seizures in the form of epilepsy in five out of their 38 patients. However, the nature of these seizures was not described in detail, and there was no indication of dysautonomia [12].

Regarding the olfactory symptoms, although hallucinations have not been reported to our knowledge, there is literature supporting the incidence of anosmia and nasal congestion. The literature review by Gopakumar et al. identified just two out of 58 cases located in the olfactory groove, one in particular resulting in anosmia [7]. This case featured a 33-year-old female who also presented with progressive headaches similar to our patient, in addition to unique symptoms such as ataxia, diminished sense of taste, and visual field deficits [14]. The reports of anosmia in a case of SFT on the olfactory groove imply that our patient’s olfactory hallucinations may be the result of direct olfactory system compression as opposed to a sequela of his supposed focal seizures in the setting of dysautonomia. Regardless of the mechanism, SFT presenting with olfactory hallucinations appears to be a novel finding.

## Conclusions

The features of this case that differentiate itself from the existing literature support a growing consensus that intracranial SFTs have a high degree of variability in presentation that is yet to be fully characterized. Presenting symptoms of olfactory hallucination and dysautonomia resulting from focal seizures in a 21-year-old patient simply adds to the novelty of an already rare diagnosis. The rates of preoperative meningioma misdiagnosis discussed above and reflected in our case underscore the importance of detailed pathologic analysis of the specimen due to the increased malignant potential of SFT. For our patient, pathologic confirmation of SFT led to further intervention in the form of adjuvant proton beam radiation and further staging with a PET scan. We hope this case contributes to the literature as the field continues to further characterize the epidemiology and possible risk factors for this potentially malignant tumor that masquerades as meningioma, requiring more extensive treatment and follow-up to mitigate the risk of recurrence or metastasis.
